# Case Report: Occupation Radiation Disease, Skin Injury, and Leukemia After Accidental Radiation Exposure

**DOI:** 10.3389/fpubh.2021.657564

**Published:** 2021-05-12

**Authors:** Xiaoji Hao, Anfang Ye, Shunfei Yu, Qianying Ni, Jiadi Guo, Xiangguo Wang, Shenyong Gao, Zhongjun Lai, Yaoxian Zhao, Zhiqiang Xuan

**Affiliations:** ^1^Department of Occupational Health and Radiation Protection, Zhejiang Provincial Center for Disease Control and Prevention, Hangzhou, China; ^2^Department of Occupational Medicine, The First Affiliated Hospital, Zhejiang University School of Medicine, Hangzhou, China

**Keywords:** ionizing radiation, acute radiation disease, radioactive tumor, leukemia, case report

## Abstract

**Objective:** Follow-up observation of radiation accident in which a worker developed acute radiation disease and eventually died of leukemia. The case provided key practical information for the study on clinical effects of radiation on the health of workers.

**Case Presentation:** We observed and followed-up the progression and effect of radiation exposure at various stages in a 28-year-old male patient. We examined the chromosomal morphology, white blood cell count, and sperm count. Laboratory tests for leukemia diagnosis and other clinical parameters were performed.

**Results:** After the patient was irradiated, the white blood cell level decreased, the sperm count dropped to 0, and the libido completely disappeared. The patient's chromosome aberration cell rate and total chromosome aberration cell rate were 7.33 and 7.66%, respectively. Examination of leukemia diagnostic experiments revealed that abnormal cells accounted for 60%; bone marrow examination showed that prolymphocytes abnormally proliferated, accounting for 89%, and had positive extracellular iron staining. After the initial treatment, the patient's white blood cell level increased and was finally maintained at a normal level, the sperm count returned to normal levels, and libido was restored. The patient died of acute lymphoblastic leukemia 34 years after the exposure.

**Conclusion:** More attention has been paid to the long-term effects of ionizing radiation-induced malignant tumors. The occupational protection of radiographic inspection workers should be strengthened to reduce and avoid occupational injuries to protect the health and safety of workers.

## Introduction

Application of ionizing radiation to an organism can produce biological effects of radiation on the organism (1). The mechanism of action can be divided into stochastic effect and deterministic effect, also called organizational response. Stochastic effects include cancers in exposed individuals due to somatic cell mutations and genetic effects in offspring due to germ cell mutations. Ionizing radiation induces malignant tumors, which has been confirmed in a large number of animal experiments and human epidemiological studies (2). However, the genetic effect induced by ionizing radiation is still lacking epidemiological evidence, and such effect has only been found in animal experiments (2). Leukopenia, cataracts, erythema depilation, and other radiation skin damages caused by ionizing radiation are all deterministic effects. In 1978, an X-ray inspection worker in Zhejiang Province, China, was accidentally exposed to an uneven systemic irradiation, resulting in acute radiation disease of the bone marrow combined with local acute radiation skin injury. Thirty-four years after the accident, the patient died of acute lymphoblastic leukemia. The case is reported as follows.

## Case Presentation

### Occupational Exposure History

A male patient born in February 1950 began work with the X-ray inspection of metal parts in 1974. In the middle of the night on December 1978, he slept at the back of the X-ray flaw detector without knowing that his colleague had started up the industrial flaw detector compartment in the operating room. The exposure time was about 30 min. After the accident site simulation, the physical dose was estimated using LiF thermoluminescence dosimeter. When exposed to light from the 2005 X-ray flaw detector produced by Shanghai Flaw Detector Factory, with a maximum capacity of 7.5 kVA, the working voltage of the normal flaw detection operation was 160 kV. The average absorbed dose rate was 0.87 Gy/min at 0 m away from the probe window (close to the probe mirror); the absorbed dose of his back skin measured by the thermoluminescence dosimeter was 26.0 Gy. The dose-effect estimation curve of chromosome aberration (3) established in our laboratory was used to estimate the systemic absorbed dose of 1.78 Gy by chromosome aberration analysis of peripheral blood lymphocytes.

### Clinical Manifestations

The patient developed nausea and vomiting once (4 h after exposure) and then had initial reactions such as generalized fatigue, dizziness, insomnia, dreaminess, and decreased appetite. Fifteen days after exposure, blisters appeared on the right side of the 6–10 thoracic vertebrae on the back, which developed from spot-like to large flaps (7 × 8 cm, equivalent to the radiation outlet of the detector probe). In addition, blisters appeared on the skin from the third intercostal to the junction of sternal body, and white scars left after 22 days; the blisters busted and formed ulcers. Furthermore, his sexual desire was significantly reduced. However, within 60 days after irradiation, there was no obvious infection or bleeding in the whole body except at the site of the radiation ulcer.

Nineteen months after irradiation, the patient was hospitalized for 1 month. After active symptomatic and supportive treatment, his general condition improved; however, the ulcers on his back did not heal. The local skin remained unhealed with conservative treatment for 3 years after exposure. In the third year, skin grafting was performed and the ulcer healed. Follow-up observation for 30 years showed no local skin ulceration or cancer. The patient was hospitalized in February 2012 (nearly 34 years after the exposure) with symptoms of dizziness, fatigue, chest tightness, and shortness of breath without obvious inducement. After comprehensive examination and analysis, the patient was clinically diagnosed with acute lymphocytic leukemia (L2 type with POSITIVE CD13/CD33). After two rounds of chemotherapy, the patient died of pulmonary infection due to bone marrow suppression on April 15.

### Laboratory Examination

**Figure 1:**
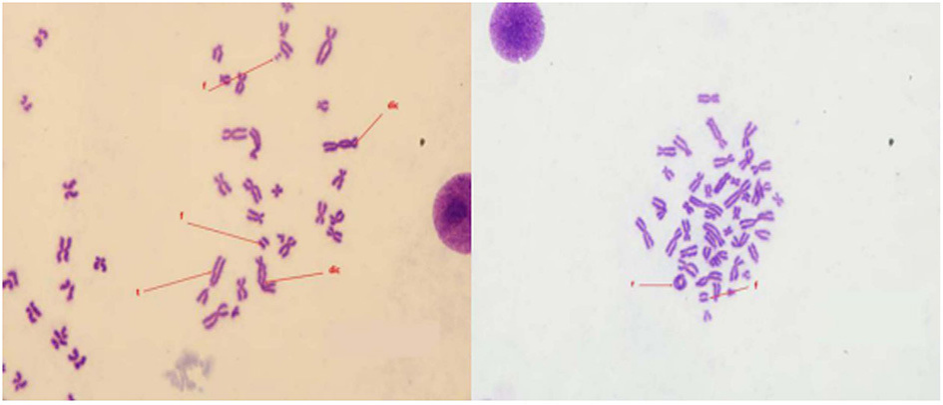
Image of chromosome aberration (Fragment [F], Translocation [T], Dicentric [dic], Centric ring [r]).

The patient's white blood cells decreased to 1.5 × 10^9^/L, 20 days after exposure and fluctuated between (1.5 and 2.4) × 10^9^/L for 18 months. Thus, the patient was hospitalized 19 months after exposure and treated with drugs to improve the leukocyte count, which increased to (4.2–5.4) × 10^9^/L. Follow-up observation showed that the white blood cells remained at the normal level. The patient's early bone marrow smear examination showed granulocyte maturation disorder but normal hematopoietic function was restored after 2 years. Eighteen months after exposure, chromosomal aberration analysis of 600 cells in the patient showed 31 double centromeres, 5 translocations, 5 centromeres, 13 pairs of microsomes, and 13 pairs of fragments, as shown in Figure 1. In the detection of chromosome aberration in peripheral blood lymphocytes, the rate of chromosome aberration at 18 months, 5, 10, 20, and 34 years after exposure was 7.3, 5.5, 0.5, 0.5, and 0%, respectively. The semen examination of the patient showed that his sperm count was 0 within 18 months after the irradiation, and sexual desire completely disappeared; at the 24th month, his sperm count was 0–1/ml; and after 26 months, his sperm count returned to the normal level, and sexual desire resumed, as shown in Table 1. In the same year, his wife became pregnant and gave birth to a normal baby girl. Follow-up observation showed that the baby girl developed well.

**Table 1:** Cell rate of semen and chromosome aberration at different times after irradiation.

	**18 months**	**24 months**	**26 months**	**5 years**	**10 years**	**20 years**	**34 years**
Sperm count (10,000/ml)	0	0–0.0001	4,600	8,400	7,600	6,800	7,100
Chromosome aberration^†^	7.33			5.5	0.5	0.5	0
Cell rate (%)							
Total chromosome^†^	7.66			6.0	1.0	0.5	0
Aberration cell rate (%)							

† *chromosome aberration tests were not performed at 24 and 26 months after the exposure*.

**Figure 2:**
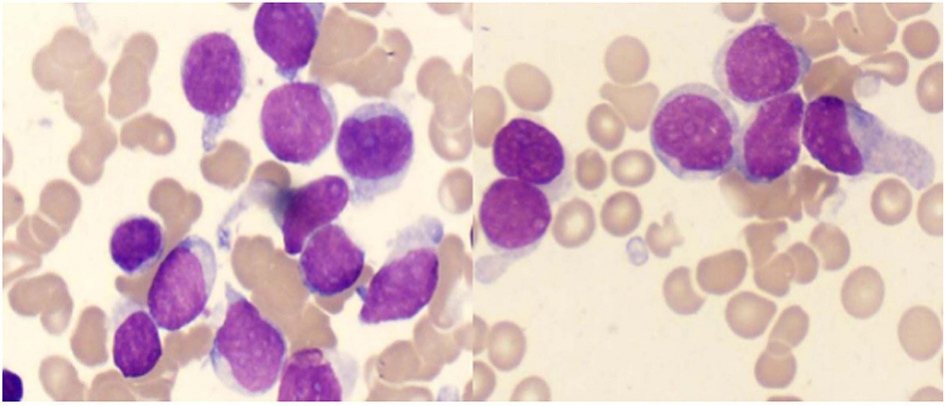
Image of the bone marrow in acute lymphoblastic leukemia.

Leukemia diagnostic test was performed and the results of blood routine showed that white blood cells (7.0 × 10^9^/L), hemoglobin (75 g/L), platelets (10 × 10^9^/L), and abnormal cells were 60%. Furthermore, bone marrow examination showed abnormal proliferation of proto-lymphocytes (89%) and positive extracellular iron staining, as shown in Figure 2. The immune types of leukemia were CD7^+^, CD13^+^, CD33^+^, CD34^+^, CD38^+^, CD45^+^, and CD117^+^. The original T-lymphocyte population accounted for 81.13% of non-erythroid. No abnormality was found in the karyotype analysis of bone marrow cells. Qualitative tests for the c-KIT/D816V gene mutation, CEBPA gene mutation, and fusion gene screening were all negative. No obvious abnormalities were found in biochemical indexes, tumor markers, B-mode ultrasound, and CT (Computed Tomography) examination.

### Calculation of the Probability of Tumor Causation

According to the current Standard for the Judgment of Occupational Radiation Tumors (GBZ 97-2017) (4), this case of malignant tumor has been included in the list of malignant tumors calculated in Table B.1 in Appendix B to calculate the probability of etiology. According to the patient's gender, the systemic absorbed dose of acute X-ray radiation at age 28 years was 1.78 Gy. After 34 years (62 years old), considering the diagnosis of acute lymphocytic leukemia and other data, the calculated PC (Probability of causation) value of the cause of cancer was 81%. The PC 95% upper limit was 200.88%, the adjusted PC' 95% upper limit was 144.93%, and the calculated 95% credible upper limit of etiology probability was > 50%, which can be judged as occupational radiation tumor.

### Cases of Diagnosis

Combined with the initial clinical manifestations, laboratory examination, dose estimation, and other data, the patient was diagnosed as acute radiation skin injury grade IV with mild systemic myeloid acute radiation disease. Moreover, 34 years after the exposure, the patient was diagnosed with acute lymphoblastic leukemia caused by excessive exposure to ionizing radiation.

## Discussion

The accidental irradiation caused the patient to receive a dose of 1.78 Gy. According to the national standard Diagnosis of Acute Radiation Sickness of Occupational External Radiation (GBZ 104-2017), although the white blood cells once dropped to 1.5 × 10^9^/L after exposure, but combined with clinical manifestations, there were no multiple vomiting and only one vomiting in the early stage, with nausea, fatigue, decreased appetite, dizziness, headache, insomnia, and other symptoms. There were no typical symptoms of fever, infection, or bleeding at the extreme stage; therefore, the patient was eventually diagnosed with mild bone marrow acute radiation disease. The leucocytopenia in this case lasted for 19 months, which may be related to the delayed leucocytopenia caused by a large skin ulcer on the back that worsened the systemic condition and delayed the recovery of white blood cells. The impact of the recovery of fertility after the exposure on the offspring is a matter of concern (5). This patient's sperm count returned to normal and he became fertile 26 months after the exposure.

Although radiation does not play a prominent role in the etiology of all human cancers like chemical carcinogens, but since nuclear technology is more and more widely used in human production and life, the potential harm of radiation to humans is also increasing (6). The proportion of radioactive tumor in occupational radiation disease is increasing year by year. Due to the high incidence of radiation-induced leukemia and a long incubation period, it is receiving increasing attention (7). Radiation-induced leukemia was first reported in Japan. The leukemia started about 2 years after the exposure and reached its peak 6–8 years after the exposure. Among the various types of leukemia induced, acute granulocytes, lymphocytic leukemia, and chronic myelogenous leukemia were the major ones (7, 8). Leukemia is one of the earliest cancer effects after acute exposure to relatively high doses of ionizing radiation (9). This case was caused by uneven radiation of the whole body, resulting in local radioactive skin damage of grade IV with mild bone marrow acute radiation sickness. In our case, acute lymphocytic leukemia was diagnosed 34 years after the exposure. To calculate the cause of cancer caused by the previous exposure, the upper limit of 95% confidence limit of PC after correction was 144.93%.

This patient developed leukemia 34 years after the exposure and experienced a long incubation period. The pathogenesis of leukemia may be different from that of total body irradiation. The skin of the patient's back was close to the X-ray detector probe for 30 min and was subjected to a large dose of local irradiation, resulting in penetrating injury from the skin of the back to the skin of the front chest. This resulted in local skin damage, as well as damage to the internal organs and hematopoietic tissues at the site. The gene mutation and chromosome aberration caused by DNA damage induced by ionizing radiation aggravated the instability of the cell genome that led to the loss of the normal growth regulation function of cells and promoted the malignant transformation of cells (10). Relevant studies have shown that ionizing radiation can lead to cluster damage of DNA molecules and generate secondary DNA damage with the action of free radicals, which is difficult to repair, has a high error repair rate, and produces far-reaching biological effects (11, 12). The induction of chromosomal aberrations in human lymphocytes exposed to ionizing radiation provides a commonly used and quantifiable biological dosimeter that can reliably estimate the radiation dose to which people are exposed (13). In this case, chromosome aberration in peripheral blood lymphocytes was still detected within 20 years of follow-up after the exposure, and the distortion rate was maintained at 0.5%. The authors believe that regular medical follow-up is necessary for patients with acute radiation disease, especially for patients with continuous positive chromosome aberrations, and the possibility from aberrations and mutations to canceration cannot be ruled out. At the same time, in order to prevent the occurrence of similar accidents, radiological protection rules and regulations should be strictly observed in the workplace, to enhance the awareness of personal protection. Workers using radiographic detection should wear personal alarm devices, to detect accidental exposures in time, reduce and avoid occupational injuries, and protect the health and safety of workers.

## Data Availability Statement

The raw data supporting the conclusions of this article will be made available by the authors, without undue reservation.

## Ethics Statement

The studies involving human participants were reviewed and approved by The Institutional Ethics Committee of the First Affiliated Hospital of Zhejiang University School of Medicine. The patients/participants provided their written informed consent to participate in this study.

## Author Contributions

ZX directed the writing and revision of the paper. SG and XW conceived ideas. XH conducted the analysis and wrote the manuscript in collaboration with AY and SY. JG, QN, ZL, and YZ provided the feedback and suggestions. All authors read the manuscript and agreed to submit it.

## Conflict of Interest

The authors declare that the research was conducted in the absence of any commercial or financial relationships that could be construed as a potential conflict of interest.
