# Supplementary material for: Case Report: Occupation Radiation Disease, Skin Injury, and Leukemia After Accidental Radiation Exposure
Source: Front Public Health. 2021 May 12;9:657564. doi: 10.3389/fpubh.2021.657564 (PMC8149743; doi:10.3389/fpubh.2021.657564)
Supplement: Supplementary file 1 [file Table_1.DOCX]

**CARE checklist**

| Topic | Item | Checklist item description | Reported |
| --- | --- | --- | --- |
| Title | 1 | The diagnosis or intervention of primary focus followed by the words “case report” | Yes ☑ No |
| Key Words | 2 | 2 to 5 key words that identify diagnoses or interventions in this case report, including "case report" | Yes ☑ No |
| Abstract  (no references) | 3a | Introduction: What is unique about this case and what does it add to the scientific literature? | Yes ☑ No |
|  | 3b | Main symptoms and/or important clinical findings | Yes ☑ No |
|  | 3c | The main diagnoses, therapeutic interventions, and outcomes | Yes ☑ No |
|  | 3d | Conclusion—What is the main “take-away” lesson(s) from this case? | Yes ☑ No |
| Introduction | 4 | One or two paragraphs summarizing why this case is unique (may include references) | Yes ☑ No |
| Patient Information | 5a | De-identified patient specific information | Yes ☑ No |
|  | 5b | Primary concerns and symptoms of the patient | Yes ☑ No |
|  | 5c | Medical, family, and psycho-social history including relevant genetic information | Yes ☑ No |
|  | 5d | Relevant past interventions with outcomes | Yes ☑ No |
| Clinical Findings | 6 | Describe significant physical examination (PE) and important clinical findings | Yes ☑ No |
| Timeline | 7 | Historical and current information from this episode of care organized as a timeline | Yes ☑ No |
| Diagnostic  Assessment | 8a | Diagnostic testing (such as PE, laboratory testing, imaging, surveys) | Yes ☑ No |
|  | 8b | Diagnostic challenges (such as access to testing, financial, or cultural) | Yes ☑ No |
|  | 8c | Diagnosis (including other diagnoses considered) | Yes ☑ No |
|  | 8d | Prognosis (such as staging in oncology) where applicable | Yes ☑ No |
| Therapeutic  Intervention | 9a | Types of therapeutic intervention (such as pharmacologic, surgical, preventive, self-care) | Yes ☑ No |
|  | 9b | Administration of therapeutic intervention (such as dosage, strength, duration) | Yes ☑ No |
|  | 9c | Changes in therapeutic intervention (with rationale) | Yes ☑ No |
| Follow-up and  Outcomes | 10a | Clinician and patient-assessed outcomes (if available) | Yes ☑ No |
|  | 10b | Important follow-up diagnostic and other test results | Yes ☑ No |
|  | 10c | Intervention adherence and tolerability (How was this assessed?) | Yes ☑ No |
|  | 10d | Adverse and unanticipated events | Yes ☑ No |
| Discussion | 11a | A scientific discussion of the strengths AND limitations associated with this case report | Yes ☑ No |
|  | 11b | Discussion of the relevant medical literature with references | Yes ☑ No |
|  | 11c | The scientific rationale for any conclusions (including assessment of possible causes) | Yes ☑ No |
|  | 11d | The primary “take-away” lessons of this case report (without references) in a one paragraph conclusion | Yes No ☑ |
| Patient Perspective | 12 | The patient should share their perspective in one to two paragraphs on the treatment(s) they received | Yes ☑ No |
| Informed Consent | 13 | Did the patient give informed consent? Please provide if requested | Yes ☑ No |
